# Does weight lifting improve visual acuity? A replication of Gonzalo-Fonrodona and Porras (2013)

**DOI:** 10.1186/s13104-017-2699-1

**Published:** 2017-08-01

**Authors:** Fumiya Yonemitsu, Yubin Sung, Kyoko Naka, Yuki Yamada, Fernando Marmolejo-Ramos

**Affiliations:** 1 Graduate School of Human-Environment Studies, Kyushu University, 6-19-1 Hakozaki, Higashi-ku, Fukuoka, 812-8581 Japan; 2 Faculty of Arts and Science, Kyushu University, 744 Motooka, Nishi-ku, Fukuoka, 819-0395 Japan; 3 School of Psychology, The University of Adelaide, Adelaide, SA 5005 Australia

**Keywords:** Embodied cognition, Neuroscience, Cross-modality

## Abstract

**Objective:**

A physical effort such as lifting up a weight affects our perception and cognition. A previous study reported in two experiments that weight lifting improves visual acuity. In the previous study, participants’ visual acuity was higher while lifting weights than while resting. Moreover, via a case study, that study further showed that the heavier the weight, the better the visual acuity. These experiments, although interesting, lacked methodological details and thorough statistical analyses. We thus conducted experiments similar to these two previous ones that mitigated these issues.

**Results:**

Although our results of Experiment 1 echoed those of the previous study, the results of Experiment 2 did not support the latter case report. Thus, our results suggest that the bodily experience of weights improves visual acuity, but a gradual increase in weight does not seem to lead to a gradual increase in visual acuity.

**Electronic supplementary material:**

The online version of this article (doi:10.1186/s13104-017-2699-1) contains supplementary material, which is available to authorized users.

## Introduction

Embodiment researchers have repeatedly shown that our bodily experience of weights influences judgment, decision making and perception [1–4]. These studies suggest that sensorimotor cues are used for adaptive behavior, cognition and perception.

Interestingly, a recent study examined whether bodily experience of weight was involved with visual acuity [5]. In the previous study, two experiments were conducted. Their Experiment 1 measured the visual acuity of 10 participants while they held weights in their hands (28 kg for males and 18 kg for females) and while they were resting without weights. The results showed that visual acuity was better when the participants held the weights than when they did not. Additionally, in their Experiment 2 weights were presented in an increasing fashion, from 1 to 15 kg, and the results showed a positive correlation between the degrees of the weights and visual acuity. These results suggest that static muscular effort improves visual acuity. Based on these findings the authors argue that, as suggested in clinical reports by Gonzalo [6, 7], basic biological scaling power laws go hand in hand with a mass activation of the neural network.

However, the study by Gonzalo-Fonrodona and Porras [5] has some methodological issues that need to be addressed. Firstly, no statistical analysis was performed, so the validity of the evidence was not ensured. Secondly, the authors themselves participated in the experiment. Thus, a kind of expectancy effect or a response bias may have contaminated the results of the previous study. Finally, the sample size of the previous study was only 10 participants and it consisted of six males and four females. This sample size was small and gender ratio was unbalanced. In order to make the results more reliable and draw an appropriate conclusion, it is essential for the current study to address these points.

Thus, in the present study we investigated whether weight lifting improves visual acuity, taking the issues mentioned above into account. Here we implemented rigorous statistical methods to analyze the data.

## Main text

### Experiment 1

#### Methods

##### Participants

Twenty Japanese people participated in Experiment 1 (10 men; overall mean age = 40.25, *SD* = 12.13, age range 22–60; mean age of men = 40.20, *SD* = 12.16; mean age of women = 40.30, *SD* = 12.13). Ten participants were recruited for Experiment 1 in the previous study (6 men; overall mean age = 38.50, *SD* = 11.16, mean age of men = 34.17, *SD* = 8.39, mean age of women = 45.00, *SD* = 11.64; age range 21–61). Participants had normal or corrected to normal visual acuity of 20/20 or better, were fully unaware of the purpose of the experiment and gave written informed consent.

##### Apparatus and stimuli

A visual acuity test was conducted via the Freiburg visual acuity and contrast test [8, 9]. The acuity stimuli consisted of two short vertical lines slightly misaligned in the horizontal direction, presented on a gray background. The size of each line was 2.5 cm and black in color. Two cloth bags that contained 1 kg dumbbells as weights to lift were used. The lifting weight was 28 kg for males and 18 kg for females (see Additional file 1).

##### Procedure

**Fig. 1:**
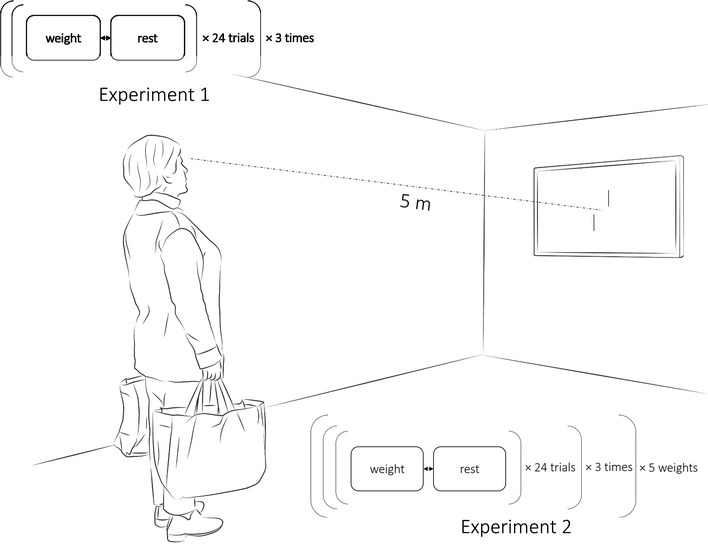
Illustration of the experimental setup

Participants looked at the monitor from a distance of 5 m and answered verbally whether the lower of the two lines was misaligned to the right or left side of the upper line. The experimental session consisted of a weight condition and a rest condition. In the weight condition, male participants had the cloth bag with weights of 14 kg in each hand, whereas female participants had the cloth bag with weights of 9 kg in each hand. In the rest condition, participants responded to the visual test without holding weights. In both cases, participants held their hands down. Each condition consisted of 24 trials and the two conditions were further repeated three times. Thus, the total number of trials was 144 (72 trials in each condition). To avoid habituation to weight and fatigue, the weight and rest conditions were alternated within participants. They were given a break for approximate 2–3 min every time they finished the two conditions. The first condition presented was counterbalanced between participants (see Fig. 1).

##### Design and statistical analyses

Data were first screened for normality and outliers. Subsequently, tests of location and scale were applied. Data were further examined via distributional analyses and linear models (see Additional file 1).

#### Results and discussion

##### Data of the previous study

The location and scale tests showed that the two conditions’ means and variances differed (*M*_*weight*_ = 13.08, *SD* = 7.10, *var* = 50.41; *M*_*rest*_ = 20.48, *SD* = 10.05, *var* = 101.17). Distributional analyses indicated the two distributions were not equal. The linear model confirmed a difference between conditions’ means. The model further indicated a significant effect of gender and a non-significant effect of age. A significant interaction between gender and condition in the model suggested that males had higher acuity (i.e. lower acuity scores) than females (*M*_*males*_ = 10.12, *SD* = 8.90; *M*_*females*_ = 24.15, *SD* = 9.68) (see Additional file 1).

##### Data of the present study

As data did not distribute normally, some observations needed to be excluded. Via bootstrap and permutation techniques, it was determined that the two groups’ means did not differ (*M*_*weight*_ = 18.14, *SD* = 7.23, *var* = 52.35; *M*_*rest*_ = 18.99, *SD* = 10.78, *var* = 116.23; p value ≈ .8). These techniques also showed that the variance ratio between the two conditions (i.e. 2.22) was significantly larger than one. The linear models did not show any significant main effects and interactions (see Additional file 1).

**Fig. 2:**
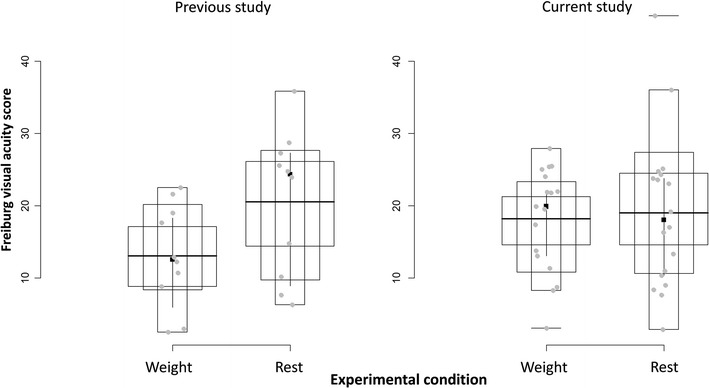
Shifting boxplots representing the normalized data in the previous and current experiments (see Additional file 1 for details)

While the results of the analyses indicate the data of the previous and present studies disagree with regard to differences between the two conditions’ means, they agree in that the two conditions differ in terms of variance (see Fig. 2). Specifically, both data sets suggest that variability in visual acuity is lower under muscular effort conditions (i.e. the weight condition) compared with that under no muscular effort (i.e. the rest condition). In other words, the variance in visual acuity is approximately 2 times (2 in the previous data and 2.22 in our data) smaller under muscular effort than under no effort.

### Experiment 2

The second experiment in the previous study [5] manipulated weights from 1 to 15 kg and found a positive correlation between the increase in weight and the increase in visual acuity. In our Experiment 2, we used a condition similar to it to confirm the effect of weight lifting on visual acuity at the individual level.

#### Methods

##### Participants

Two Japanese males participated in Experiment 2 of this study. One of the participants (participant U) was 23 years old, and the other (participant V) was 44 years old. Note that the only participant in Experiment 2 of the previous study was a 45-year-old Spanish male (participant H). These two had normal or corrected to normal visual acuity of 20/20 or better, and were fully unaware of the purpose of the experiment and gave written informed consent.

##### Apparatus, stimuli and procedure

The procedure of Experiment 2 was identical to that of Experiment 1 in the present study. However, there was an increase in the weights as reported in Gonzalo-Fonrodona and Porras’s [5] case report. As in Experiment 1, a session consisted of a weight condition and a rest condition, had 24 trials in each condition, and was repeated three times. The weights held by participants were increased in the order of 1, 2, 3, 5 and 15 kg in each hand at the end of each of the three repetitions. The total number of trials was 720 (360 trials per condition).

##### Design and statistical analyses

The relationship between increase in weight and, potentially, decreases in visual acuity scores (i.e. increase in visual acuity) were assessed via correlation tests (see Additional file 1).

#### Results and discussion

##### Data of the previous study

The results indicated a negative association between weight and visual acuity scores; i.e. the higher the weight, the lower the acuity score (i.e. higher the visual acuity) (*r*_*S*_ = −.95, *p* = .001).

##### Data of the present study

The results did not suggest any association between weight and visual acuity (participant U: *r*_*S*_ = .08, *p* = .91; participant V: *r*_*S*_ = .77, *p* = .10).

**Fig. 3:**
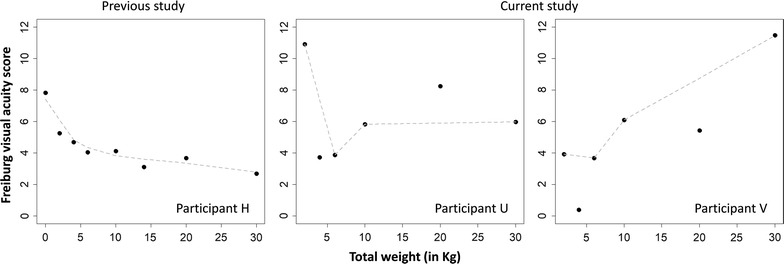
Scatterplots of the association between weight and visual acuity in the previous and current experiments (see Additional file 1 for details)

In Experiment 2, the re-analysis of the previous study’s data confirms the findings by Gonzalo-Fonrodona and Porras [5], but the result in the current experiment did not support the effect reported by them (see Fig. 3). We believe this disagreement in results is due to individual differences and the number of trials. As reported by Abbud and Cruz [10], in order to attain an average acuity value with a 10% precision, between 100 and 700 trials are needed. In the current study, each participant underwent 360 trials in the weight condition, a number that sits in the mid to low end of the range suggested. Abbud and Cruz also acknowledge that inter-individual variability is a factor that hinders definite results.

#### Conclusions

The experiments reported herein aimed at investigating whether weight lifting improves visual acuity as reported in Gonzalo-Fonrodona and Porras [5]. While the first experiment found no differences between the weight and the rest conditions’ visual acuity means, it did coincide with the previous study in that variance in acuity scores reduced during muscular effort conditions. Interestingly, it was found that variance under these conditions was unique to the present study. The general message from both the previous and current experiments is that the data in both experimental conditions are not equal. Our second experiment did not support the results exhibited by participant H in the study by Gonzalo-Fonrodona and Porras and any similarity to what was observed by those authors is merely anecdotal.

In conclusion, we tested the finding that being subjected to muscular effort leads to higher visual acuity than being in resting conditions. This claim is based on a distributional analysis that shows that the distributions of data in both conditions are not equal. However, we believe that differences in variance (rather than location) between the two experimental conditions can be a distinctive marker of the effect studied. As to the issue of the data’s scale, we would like to add that statistical analyses should not only focus on location parameters, but take into account information about the data’s scale and shape.

## Limitation

Several methodological aspects could have played part in the results obtained. As indicated by Abbud and Cruz [10], many trials are needed to dampen the effect of intra-individual variability. We strongly believe this is an issue that future studies should address. Additionally, our participants were not tested monocularly (which seems to be the usual way visual acuity is performed), but binocularly. The distance between the participant and the screen was 5 m but other authors have used longer distances (e.g. Abbud and Cruz used 10.73 m). This might be another factor to consider for future studies. Although the analyses of the previous and present studies’ data did not show an effect of age, age has been shown to influence acuity scores (see [10]). Gender seems to play part in the effect too, as suggested by the re-analysis of the data collected by Gonzalo-Fonrodona and Porras [5]. In our data, the effect of gender was not evident; however, gender effects are known to occur in neuropsychological studies. Moreover, participants in both the present and previous studies held no object in the control condition. Finally, it could be entertained that simple sensory stimulus in the hands, and not weight itself, might have influenced visual acuity. These are potential factors that need to be accounted for in forthcoming studies.

## Additional file

**Additional file 1.** Methodological details of Experiment 1 and 2 are described.
