# Supplementary material for: Does weight lifting improve visual acuity? A replication of Gonzalo-Fonrodona and Porras (2013)
Source: BMC Res Notes. 2017 Aug 1;10:362. doi: 10.1186/s13104-017-2699-1 (PMC5540219; doi:10.1186/s13104-017-2699-1)
Supplement: Supplementary file 1 — Additional file 1. Methodological details of Experiment 1 and 2 are described. [file 13104_2017_2699_MOESM1_ESM.pdf]

Methodological details

Experiment 1

Apparatus and Stimuli: the experiment was conducted in a dark room. All stimuli were presented on a 60-inches Pioneer PDP-607CMX Plasma Display (refresh rate = 59.9 Hz; resolution = 1376 × 768).

Design and statistical analyses: Both the data from the previous study [1] and this study were analyzed. First, normality (via the Shapiro-Wilk normality test [2]) and outlier checks (via boxplots) were performed. Tests of location and scale were carried out via a bootstrap percentile *t* method (see [3]; implemented in R via the function 'trimcibt') and a heteroscedastic version of the Morgan-Pitman test (see [4]; implemented in the R function 'comdvar'), respectively. The *p*-values associated with the tests of location and scale were combined via the Stouffer method to determine the equality of two distributions [5]. Finally, an automated mixed ANOVA modelling method called aLMM (see [6]; implemented in the function 'lmer' in the 'lmerTest' R package) was carried out. The fixed factors were gender (i.e. male; female), condition (weight and rest conditions) and age; the random factors were participant, number of trials (24 per condition) and repetitions (three repetitions of the 24 trials in each condition), and the dependent variable was the visual acuity scores. The model assessed was: $\mathrm{s} = \mathrm{g} + \mathrm{c} + \mathrm{g}\times\mathrm{c} + p + t + r$; where s=visual acuity scores, g=gender, c=condition, p=participant, t=number of trials, and r=repetitions. The shifting boxplot was used to show data's distribution [7]. In these plots, observations lying ±2 SD beyond the mean are represented by dashes, observations between −2 and +2 SD are the longest and thinnest boxes, observations that fall between the mean of the first half of the data and the mean of the second half of the data are the intermediate boxes, the means of the data are the middle thickest and longest horizontal lines, and their 95% bootstrap bias-corrected and accelerated confidence intervals are the outermost shortest and thickest boxes. Also, the medians and their 95% bootstrap CIs are represented by a solid small square and whiskers around them. Participants' mean acuity scores are shown as filled grey circles.

Results

Data of the previous study: The normality tests indicated that the acuity scores in the weight and rest conditions were distributed normally (W_{weight} = .93, *p*-value = .51; W_{rest} = .91, *p*-

value = .33), and the boxplots did not signal any outliers. The location and scale tests showed that the two conditions' means and variances differed (M_{weight} = 13.08, SD = 7.10, var = 50.41; M_{rest} = 20.48, SD = 10.05, var = 101.17) such that the p-values associated with their comparisons were .025 (difference in location) and .011 (difference in scale). The combined p-values of these tests indicated the two distributions were not equal (p-value$_{Stouffer}$ = 1.81e^{-73}). The results of the aLMM confirmed the differences between conditions' means by a main effect of condition ($F(1, 78.16) = 51.53$, $p < 1e^{-7}$). This analysis also showed a significant effect of gender ($F(1, 7.95) = 6.46$, $p = .03$), no significant effect of age ($F(1, 6.91) = .02$, $p = .88$), and a significant interaction between gender and condition ($F(1, 78.16) = 8.42$, $p = .004$). The significant effect of gender suggested that males had higher acuity (i.e. lower acuity scores) than females (M_{males} = 10.12, SD = 8.90; $M_{females}$ = 24.15, SD = 9.68), and the significant interaction showed this pattern occurred in both experimental conditions. Out of the random effects, only the factor participants had a significant effect ($\chi^2(1) = 38.03$, $p < 1e^{-7}$; $\chi^2(1) = .14$ [number of trials]; $\chi^2(1) = .61$ [repetitions]).

Data of the present study: The normality tests and boxplots suggested data did not distribute normally (W_{weight} = .74, p-value < .001; W_{rest} = .87, p-value = .01) due to three large observations in the weight condition group and two large values in the rest condition group. After removing those observations, both distributions became Gaussian shaped (W_{weight} = .93, p-value = .23; W_{rest} = .92, p-value = .19). Eliminating observations had the effect of altering the groups' sample sizes to 17 (weight condition) and 18 (rest condition). Bootstrap and permutation simulations were used to perform pairwise comparison of location and scale of these unbalanced dependent samples. These techniques suggested that the two groups' means did not differ (M_{weight} = 18.14, SD = 7.23, var = 52.35; M_{rest} = 18.99, SD = 10.78, var = 116.23; p-value ≈ .8). By using these techniques, it was also found that the variance ratio between the two conditions (i.e. 2.22) was significantly larger than one; the 95% CI around the most likely value [8] in the bootstrapped distribution of the two groups' variance ratio did not include one (*Mode* = 1.98 [1.59, 2.11]. Indeed, a 95% CI around the distribution's 20% trimmed mean also suggested the observed variance ratio was larger than one ($M_{20\%\ trim}$ = 2.21 [2.16, 2.26]). Location and scale tests performed on the two conditions prior to outlier elimination also showed that the groups did not differ in terms of location ($t(19)=1.22$, $p=.23$), but differed in terms of scale ($t(18) = 2.38$, $p = .02$), and the combination of these p-values indicated the two distributions were not equal ($p = .031$). The aLMM analysis did not

show any significant main effects and interactions.

Experiment 2

Design and statistical analyses: Spearman rank correlations were used to investigate the relationship between increases in weight and, potentially, decreases in visual acuity scores (i.e. increase in visual acuity). Data trends were represented via robust locally weighted regression lines (LOWESS).

References

1. Gonzalo-Fonrodona, I., & Porras, M. A. Scaling effects in crossmodal improvement of visual perception by motor system stimulus. Neurocomputing. 2013;114:76-79.
2. Marmolejo-Ramos, F., & González-Burgos, J. A power comparison of various normality tests of univariate normality on Ex-Gaussian distributions. Methodology. 2013;9:137-49.
3. Wilcox, R. (2016). Introduction to Robust Estimation and Hypothesis Testing. Amsterdam: Elsevier.
4. Wilcox, R. Comparing the variances of two dependent variables. J Stat Distrib Appl. 2015;2. doi: 10.1186/s40488-015-0030-z
5. Frank, J., & Klar, B. Methods to test for equality of two normal distributions. Stat Methods Appt. 2016;25:581-99.
6. Kuznetsova, A., Christensen, R., Bavay, C., & Brockhoff, P. Automated mixed ANOVA modelling of sensory and consumer data. Food Qual Prefer. 2015;40:31-8.
7. Marmolejo-Ramos, F., & Tian, S. The shifting boxplot. A boxplot based on essential summary statistics around the mean. Int J Psychol Resy. 2010;3:37-46
8. Parzen, E. On estimation of a probability density function and mode. Ann Math Stat. 1962;33:1065-76.
